# Ferroptosis-Like Death in Microorganisms: A Novel Programmed Cell Death Following Lipid Peroxidation

**DOI:** 10.4014/jmb.2307.07002

**Published:** 2023-07-20

**Authors:** Min Seok Kwun, Dong Gun Lee

**Affiliations:** School of Life Sciences, BK 21 FOUR KNU Creative BioResearch Group, College of Natural Sciences, Kyungpook National University, Daehakro 80, Bukgu, Daegu 41566, Republic of Korea

**Keywords:** Ferroptosis-like death, reactive oxygen species, microorganisms, poly unsaturated fatty acid

## Abstract

Ferroptosis is a new kind of programmed cell death of which occurrence in microorganisms is not clearly verified. The elevated level of reactive oxygen species (ROS) influences cellular metabolisms through highly reactive hydroxyl radical formation under the iron-dependent Fenton reaction. Iron contributes to ROS production and acts as a cofactor for lipoxygenase to catalyze poly unsaturated fatty acid (PUFA) oxidation, exerting oxidative damage in cells. While ferroptosis is known to take place only in mammalian cells, recent studies discovered the possible ferroptosis-like death in few specific microorganisms. Capacity of integrating PUFA into intracellular membrane phospholipid has been considered as a key factor in bacterial or fungal ferroptosis-like death. *Vibrio* species in bacteria and *Saccharomyces cerevisiae* in fungi exhibited certain characteristics. Therefore, this review focus on introducing the occurrence of ferroptosis-like death in microorganisms and investigating the mode of action underlying the cells based on contribution of lipid peroxidation and iron-dependent reaction.

## Introduction

Under life-threatening infections from pathogens, efforts are made to seek for antibiotics which regulate the cell death of pathogenic microorganisms. Due to side effects from various antibiotics when injected into human body, wide investigations on mechanisms of microbial cell death have been continued for effective control of human inflammatory reaction and toxicity of antimicrobial agents. As a result, several regulated cell deaths are currently revealed, including apoptosis, pyroptosis and ferroptosis [1].

Programmed cell death (PCD) is interpreted as the death of any cell mediated by an intracellular program [2]. The discovery of PCD in unicellular organisms initially faced criticism and disbelief, as the phenomenon was thought to occur only in multicellular organisms [3-5]. Currently, the presence of genetically encoded cell death pathways in single cell organisms and the existence of several different PCD mechanisms in response to environmental stimuli have been described [3, 5]. The PCD system has been reported to play critical roles in bacterial pathogenesis and survival, and it has been identified in various bacterial species, such as *Mycobacterium tuberculosis*, *Staphylococcus aureus*, *Bacillus* and *Escherichia coli*, *Anabaena*, *Yersinia*, *Caulobacter*, and *Streptococcus pneumoniae* [5, 6]. Moreover, PCD has been also identified in fungal species, including *Candida albicans*, *Saccharomyces cerevisiae*. PCD in microorganisms is a promising research area that can lead to the development of innovative antimicrobial agents [7].

Recently, new type of PCD has been discovered, excluding apoptosis or autophagy; which is called “ferroptosis” and was first described by Dixon in 2012 [8]. Unlike previous PCD, ferroptosis exhibits morphological features, such as the dissipation of mitochondrial cristae, decreased mitochondrial volume, and the collapse of the outer mitochondrial membrane [9, 10]. In addition, biochemical characteristics, such as overloading of intracellular iron and oxidation of polyunsaturated fatty acid (PUFA) appear [11, 12]. The mammalian mechanism of ferroptosis is classified into two main pathways; first, the inactivation of glutathione peroxidase 4 (GPx4) due to glutathione (GSH) deficiency [13]. This is caused by a deficit of the GSH precursor, cysteine (Cys), which inactivates GPx4, an endogenous enzyme that suppresses lipid peroxidation [14]. Eventually, failure of the GSH-dependent antioxidant defense leads to the implementation of ferroptosis. The second pathway is iron accumulation [15]. Usually, iron contributes to reactive oxygen species (ROS) production and acts as a cofactor for lipoxygenase to catalyze PUFA oxidation [9, 16]. Therefore, when iron is overloaded in cells, toxic ROS are produced, and PUFA is deficient, leading to ferroptosis. Various ferroptosis inducers exist based on these mechanisms, such as erastin and sulfasalazine, which inhibit Cys uptake, or RSL-3, which inactivates GPx4 [1, 17]. Based on ferroptotic hallmarks, this review aims to introduce the occurrence of ferroptosis-like death in microorganisms with an effort to investigate their mode of action for further application of antimicrobial agents through diverse mechanisms.

## The Physiological Benefit of Maintaining PCD Mechanisms

Cellular self-destruction seems counterintuitive to evolutionary processes and the driving forces of natural selection [18]. Nowadays, many researchers believe that bacteria undergo evolutionarily conserved processes for the benefit of their kin and preservation of the bacterial population as a whole, although PCD mechanisms had been largely unexplored due to the diversity of bacteria and their varied life styles [2, 3]. Bacterial PCD functions in both the development of multicellular structures and the prolonged preservation of the bacterial population [18, 19]. For example, cells undergo the process of releasing nutrients, increasing the fitness of a population as a whole under conditions of nutrient starvation. PCD results in obtaining DNA, RNA, proteins, and other essential cellular components for the survival of other starving cells. The rest of the bacterial population benefits from the PCD of some cells [20]. The advantages of maintaining suicide genes are mostly for the whole population as opposed to the free-living individual. Thus, the genes are maintained, as evolution works on populations, not individuals [21]. For instance, restriction endonuclease (REase)-mediated cell death is caused by an apoptotic pathway and is beneficial for isogenic bacterial communities [2, 22]. The cells undergoing REase-mediated cell death release nutrients, supporting the growth of the remaining cells in the population. REase-induced PCD appears to be a cellular design for replenishing nutrients in cells undergoing starvation stress, and the phenomenon could be widespread in bacteria, given the abundance of restriction-modification (R-M) systems in the microbial population [2]. Indeed, PCD clearly contributes to evolutionarily conserved processes for population benefit [2]. The existence of an active death pathway in single-cell organisms raises evolutionary questions regarding the selective advantage of suicide following severe stress.

There is another evolutionarily conserved mechanism that mediates cell fate [23]. Aerobic organisms rely on oxygen for their energy production. Paradoxically, oxygen toxicity induces molecular damage. Antioxidant strategies have evolved to counterbalance the highly toxic oxygen derivates, commonly called ROS [23]. Through ROS-mediated post-stress self-destruction, ROS contributes to the lethality of diverse stressors [24]. ROS attacks lesions of sugars and bases, resulting in genotoxicity via oxidation or single- and double-strand DNA breaks (DSB)[25-27]. The guanine base is the main target because it has the lowest ionization potential of any DNA constituent [25, 28]. Oxidative stress is controlled by redox-active transcriptional regulators that induce the expression of protective detoxification, repair, and metabolic programs [29]. Self-destruction suggests novel ways to control bacterial populations if small-molecule adjuvants are found that stimulate ROS accumulation during antimicrobial treatment [24].

## Iron Overload and Ferroptosis-Like Death

Iron is an essential element for life such as respiration, cellular metabolism, DNA synthesis, and repair. Moreover, the iron balance remains constant by coordinated mechanisms, but iron dyshomeostasis induces cell damage or free oxygen radical production. For example, disruption of iron homeostasis is a precondition that can promote ferroptosis. However, the mechanism for why or how iron deposits cause ferroptosis is yet to be identified. Therefore, it was investigated whether AuNPs generate iron overload in *S. cerevisiae* compared with erastin, increasing the intracellular iron levels. Cells treated with AuNPs had high total iron levels to a similar extent to those treated with erastin. This finding indicated that AuNPs trigger iron accumulation within *S. cerevisiae*, which can induce ferroptosis. The iron accumulation described earlier generates OH∙, a powerful ROS, through Fenton reaction. Cys or GSH deficiency contributes to oxidative stress generation without a proper antioxidant defense system.

Reactive oxygen species (ROS) are by-product of normal oxygen metabolism and necessary for cell signaling and homeostasis. In contrast, excessive ROS is detrimental to mostly intracellular components. Extended exposure or overproduction of ROS induce the bacterial response to lethal stress [30, 31]. ROS generation is caused by hyperactivation of the respiratory electron transport chain [25]. The elevation of ROS in response to diverse stressors leads to the alternation of metabolism and the dysfunction and damage of intracellular components, contributing to cellular death [32, 33]. Three naturally occurring species- hydroxyl radical, superoxide, and hydrogen peroxide - are receiving the most attention [31].

**Fig. 1:**
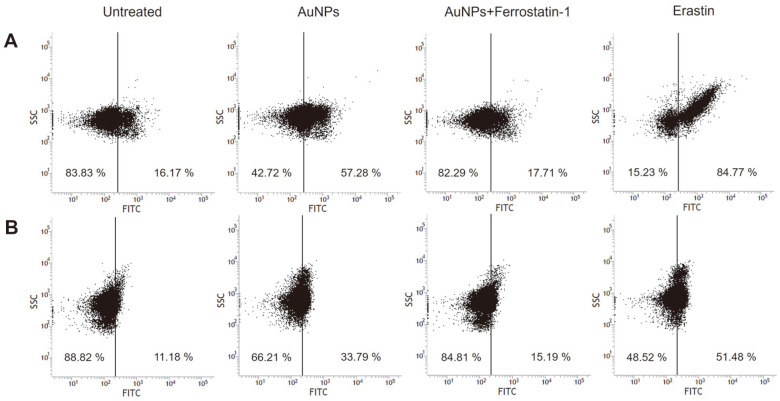
Flow cytometry analysis of hydroxyl radical generation was detected using HPF assay. (**A**) *S. cerevisiae*. (**B**) *V. vulnificus*.

When oxygen receives an electron, an instable form of superoxide anion is generated, which mostly converted to hydrogen peroxide, under the contribution of superoxide dismutase. Due to its stability, hydrogen peroxide is well-regulated in intracellular components and turns to non-toxic H2O under the control of antioxidant enzyme catalase and peroxidase. However, under excessive generation of hydrogen peroxide, the molecules receive an additional electron, which eventually forms a highly toxic molecule of hydroxyl radical [34]. Hydroxyl radical mostly accounts for the intracellular oxidative damage based on its relatively high toxicity compared to other ROS. The Fenton reaction refers to a reaction between hydrogen peroxide and ferrous ion to form ferric iron and hydroxyl radical [35]. Under the presence of ferrous ion, hydrogen peroxide and superoxide serve as substrates for highly reactive hydroxyl radical formation through the iron-dependent Fenton reaction, which can trigger lethal oxidative damage in cells, even destructing DNA. Their high toxicity prompts stress-stimulated self-destruction [31, 32]. Hydroxyl radical oxidizes lipid to induce lipid peroxidation in cells, which is an essential process in ferroptosis with presence of ferrous ion. For verification of ROS generation in ferroptosis-like death, detection of hydroxyl radical was monitored when well-known antimicrobial agent AuNP was treated in *S. cerevisiae* and *V. vulnificus* (Fig. 1).

## Glutathione, Lipid Peroxidation and Ferroptosis-Like Death

Under excessive oxidative damage accumulation, precondition for ferroptosis is GSH depletion and GPx4 inactivation due to Cys deficiency. GSH is essential to the cellular antioxidant defense; the lack of Cys inhibits intracellular GSH synthesis. Therefore, if the intracellular GSH exists below approximately 10%, the antioxidant defense system is interrupted. In addition, Cys deficiency inactivates GPx4 [14]. Since the Gpx4 gene is a ferroptosis inhibitor, disabling GPx4 indicates that ferroptosis can be initiated.

Ferroptosis is a novel type of programmed necrosis, which has a different mechanism than conventional cell death, presenting the potential to become a new immunotherapy and cancer treatment [36-38]. This ferroptotic death has been discovered not only in humans and mice, but also in yeast and bacteria [39]. However, there are certain limitations seen in eukaryotic microbes and prokaryotes regarding ferroptotic death. Because ferroptosis-related proteins, genes, and enzymes are found in yeast and bacteria, but many of their functions are unknown or unfindable.

The ferroptosis in microorganisms has long been considered as an inappropriate assume due to their structural difference to other ferroptosis-induced cells. Ferroptosis occur under the presence of PUFA which normally are not synthesized in yeast or bacterial cells. Poorly oxidizable saturated or mono-unsaturated lipid molecules are normally components of bacterial or fungal cells. While polyunsaturation is not required in most species, few exceptions exist, including marine bacteria like *Vibrio* species and fungal strain *S. cerevisiae*. The difference of these strains from other microbes is an ability to accumulate PUFA from the environment and integrate them into membrane phospholipids [39]. This characteristic enable the bacterial and fungal cell to exhibit ferroptotic hallmarks, with an possibility of discovery of new PCD in microorganisms. Although ferroptosis does not appear directly in *S. cerevisiae*, used as a model in our paper, GSH and GPx4-like protein (GPx3) exist, and it is also known that lipid peroxidation has been detected [40-42]. Therefore, our study investigated the ferroptosis-like death exhibited based on this evidence.

**Fig. 2:**
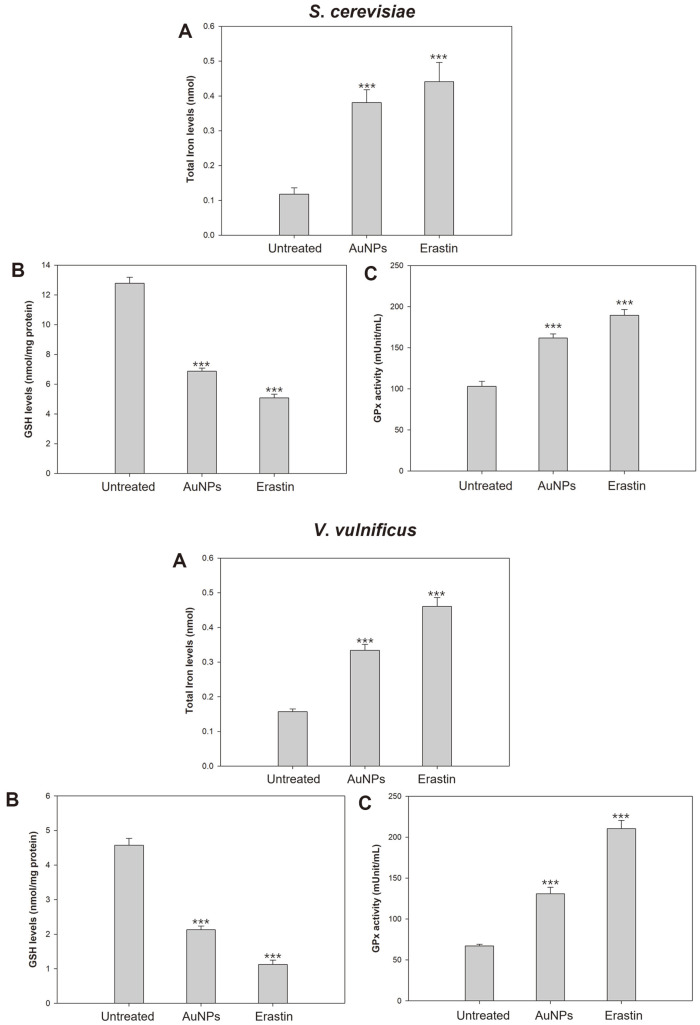
Identification of prerequisites for ferroptosis to execute. (**A**) Intracellular iron accumulation. (**B**) Intracellular glutathione (GSH) levels. (**C**) Glutathione peroxidase (GPx) activity. Experiments were conducted in triplicate independently, and the results represent the average, standard deviation, and p values from three experiments (*p < 0.1; **p < 0.05; ***p < 0.01 vs. untreated sample).

In order to verify the occurrence of ferroptosis-like death in certain species, several experiments were held under application of antimicrobial agents in fungi and bacteria. In *S. cerevisiae*, given the presence of GPx in *S. cerevisiae*, which functions as GPx4, we observed the intracellular GSH levels and activity of GPx. Consequently, both intracellular GSH levels and GPx activity decreased in AuNPs-treated cells, suggesting that AuNPs can provide pro-ferroptotic stimuli for *S. cerevisiae* due to Cys deficiency The results imply the exhibition of ferroptosis-like death hallmark in microorganisms, casting the possibility of new typed fungal PCD. Lipid peroxidation during ferroptosis is also associated with iron metabolism and GPx activity. In undamaged cells, GPx4 functions properly, preventing lipid peroxidation by the antioxidant system, but when GPx4 is inactivated, oxidation of plasma membrane lipids occurs, leading to ferroptosis. Moreover, the ROS produced by iron accumulation reacts with PUFAs, resulting in lipid peroxidation. This study detected that the MDA levels, the final lipid peroxidation product, were increased in AuNPs-treated cells (Fig. 2). In addition, we have previously identified iron accumulation and GPx inactivation, so the aggregation of these findings suggested that GPx inactivation and iron accumulation induced by AuNPs lead to lipid ROS generation and lipid peroxidation.

**Fig. 3:**
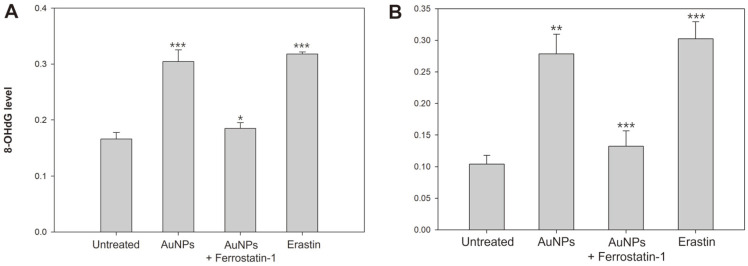
Oxidative DNA damage was performed by 8-OHdG quantitation. (**A**) *S. cerevisiae*. (**B**) *V. vulnificus*. Experiments were conducted in triplicate independently. The results represent the average, standard deviation, and *p* values from three experiments (*p < 0.1; **p < 0.05; ***p < 0.01 vs. untreated sample).

Most organisms have plasma membranes composed of a phospholipid bilayer. Therefore, lipid peroxidation due to oxidative stress causes plasma membrane damage. In detail, oxidized lipids thin thickness of cell membranes, deactivate membrane-attached enzymes, and alter the membrane structure. They also interfere with diffusion or osmosis, causing ion imbalance and eventually destroying membrane permeability. In addition, lipid peroxidation during ferroptosis elicits membrane rupture, not blisters. The oxidative stress generated by ROS attacks DNA strains and bases, making genome integrity unstable during DNA replication or repair. This genotoxic stress causes DNA damage and mutation. Guanine is a purine base paired with cytosine, and when DNA is oxidized, guanine forms 8-OHdG modification. These DNA adducts cause mutations by preventing DNA polymerase from recognizing guanine. The intracellular 8-OHdG levels were estimated to determine whether AuNPs-produced ROS oxidized the DNA of *S. cerevisiae*. As a result, AuNPs produced a significant amount of 8-OHdG and demonstrated that the AuNPs-induced oxidative stress leads to DNA damage (Fig. 3). Furthermore, it was found that AuNPs-caused iron overload and GSH deficiency generated intracellular ROS, resulting in a powerful oxidative damage to lipids and DNA.

## Conclusion and Further Remarks

The rapid rise of antibiotic resistance in pathogens is now considered a major global health crisis. New strategies are needed to block the development of resistance and to extend the life of antibiotics. Lethal concentrations of bactericidal antibiotics result in the production of harmful hydroxyl radicals through a common oxidative damage cellular death pathway that involves alterations in central metabolism. In light of the looming antibiotic crisis, such novel strategies to combat bacterial infections are desperately needed. To resolve the problem of the evolution of antibiotic resistance, a drug to modulate the PCD pathway response is going to be promising for developing therapeutics to reduce the acquisition of antibiotic resistance and enhance antimicrobial activity. Microbial ferroptotic pathway provide perspective for understanding how microorganisms deal with different stress conditions and choose their cellular fate between living and death. These new sites can provide novel targets to modulate iron overload and GPx activity in micororganisms. The accumulation of ROS leading to fenton reaction exert a lethal influence in intracellular activity, inducing lipid peroxidation which enhances DNA damage in cells. The discovery of bacterial & fungal ferroptosis are regulated under the presence of PUFA, and certain species like *V. vulnificus* and *S. cerevisiae* exhibit ferroptotic activity due to their ability of integrated PUFA into membrane phospholipids. Such mode of action can open the possibility of developing a new therapeutic strategy for potentiating antimicrobial agents that target the artificial activation of PCD. Nevertheless, given bacteria’s diversity and varied environments, unexplored PCD mechanisms might remain, and continuous research is required. Further genetic and transcriptional studies are needed to identify PCD’s operation in detail in a variety of microorganisms.

## References

[1] Yu H, Guo P, Xie X, Wang Y, Chen G (2017). Ferroptosis, a new form of cell death, and its relationships with tumourous diseases. J. Cell. Mol. Med..

[2] Nagamalleswari E, Rao S, Vasu K, Nagaraja V (2017). Restriction endonuclease triggered bacterial apoptosis as a mechanism for long time survival. Nucleic Acids Res..

[3] Dewachter L, Verstraeten N, Fauvart M, Michiels J (2016). The bacterial cell cycle checkpoint protein Obg and its role in programmed cell death. Microb. Cell..

[4] Allocati N, Masulli M, Di Ilio C, De Laurenzi V (2015). Die for the community: an overview of programmed cell death in bacteria. Cell Death Dis..

[5] Tanouchi Y, Lee AJ, Meredith H, You L (2013). Programmed cell death in bacteria and implications for antibiotic therapy. Trends Microbiol..

[6] Andryukov BG, Somova LM, Timchenko NF (2018). Molecular and genetic characteristics of cell death in prokaryotes. Mol. Genet. Microbiol. Virol..

[7] Lewis K (2000). Programmed death in bacteria. Microbiol. Mol. Biol. Rev..

[8] Dixon SJ, Lemberg KM, Lamprecht MR, Skouta R, Zaitsev EM, Gleason CE (2012). Ferroptosis: an iron-dependent form of nonapoptotic cell death. Cell.

[9] Battaglia AM, Chirillo R, Aversa I, Sacco A, Costanzo F, Biamonte F (2020). Ferroptosis and cancer: mitochondria meet the "iron maiden" cell death. Cells.

[10] Wang H, Liu C, Zhao Y, Gao G (2020). Mitochondria regulation in ferroptosis. Eur. J. Cell Biol..

[11] Chen X, Comish PB, Tang D, Kang R (2021). Characteristics and biomarkers of ferroptosis. Front. Cell Dev. Biol..

[12] Latunde-Dada GO (2017). Ferroptosis: role of lipid peroxidation, iron and ferritinophagy. Biochim. Biophys. Acta Gen. Subj..

[13] Cao JY, Dixon SJ (2016). Mechanisms of ferroptosis. Cell. Mol. Life Sci..

[14] Yang WS, Stockwell BR (2016). Ferroptosis: death by lipid peroxidation. Trends Cell Biol..

[15] Chen X, Yu C, Kang R, Tang D (2020). Iron metabolism in ferroptosis. Front. Cell Dev. Biol..

[16] Stoyanovsky DA, Tyurina YY, Shrivastava I, Bahar I, Tyurin VA, Protchenko O (2019). Iron catalysis of lipid peroxidation in ferroptosis: regulated enzymatic or random free radical reaction?. Free Radic. Biol. Med..

[17] Xie Y, Hou W, Song X, Yu Y, Huang J, Sun X (2016). Ferroptosis: process and function. Cell Death Differ..

[18] Bayles KW (2014). Bacterial programmed cell death: making sense of a paradox. Nat. Rev. Microbiol..

[19] Dewachter L, Verstraeten N, Monteyne D, Kint CI, Versees W, Perez-Morga D (2015). A Single-amino-acid substitution in Obg activates a new programmed cell death pathway in *Escherichia coli*. MBio.

[20] Kohanski MA, Dwyer DJ, Collins JJ (2010). How antibiotics kill bacteria: from targets to networks. Nat. Rev. Microbiol..

[21] Peeters SH, de Jonge MI (2018). For the greater good: programmed cell death in bacterial communities. Microbiol. Res..

[22] Mruk I, Kaczorowski T, Witczak A (2019). Natural tuning of restriction endonuclease synthesis by cluster of rare arginine codons. Sci. Rep..

[23] Schippers JH, Nguyen HM, Lu D, Schmidt R, Mueller-Roeber B (2012). ROS homeostasis during development: an evolutionary conserved strategy. Cell. Mol. Life Sci..

[24] Hong Y, Li L, Luan G, Drlica K, Zhao X (2017). Contribution of reactive oxygen species to thymineless death in *Escherichia coli*. Nat. Microbiol..

[25] Lee B, Hwang JS, Lee DG (2019). Induction of apoptosis-like death by periplanetasin-2 in *Escherichia coli* and contribution of SOS genes. Appl. Microbiol. Biotechnol..

[26] Salehi F, Behboudi H, Kavoosi G, Ardestani SK (2018). Oxidative DNA damage induced by ROS-modulating agents with the ability to target DNA: a comparison of the biological characteristics of citrus pectin and apple pectin. Sci. Rep..

[27] Srinivas US, Tan BWQ, Vellayappan BA, Jeyasekharan AD (2019). ROS and the DNA damage response in cancer. Redox Biol..

[28] Basu S, De D, Dev Khanna H, Kumar A (2014). Lipid peroxidation, DNA damage and total antioxidant status in neonatal hyperbilirubinemia. J. Perinatol..

[29] Crawford MA, Tapscott T, Fitzsimmons LF, Liu L, Reyes AM, Libby SJ (2016). Redox-active sensing by bacterial DksA transcription factors is determined by cysteine and zinc content. MBio.

[30] Dwyer DJ, Belenky PA, Yang JH, MacDonald IC, Martell JD, Takahashi N (2014). Antibiotics induce redox-related physiological alterations as part of their lethality. Proc. Natl. Acad. Sci. USA.

[31] Zhao X, Drlica K (2014). Reactive oxygen species and the bacterial response to lethal stress. Curr. Opin. Microbiol..

[32] Belenky P, Ye JD, Porter CB, Cohen NR, Lobritz MA, Ferrante T (2015). Bactericidal antibiotics induce toxic metabolic perturbations that lead to cellular damage. Cell Rep..

[33] Lobritz MA, Belenky P, Porter CB, Gutierrez A, Yang JH, Schwarz EG (2015). Antibiotic efficacy is linked to bacterial cellular respiration. Proc. Natl. Acad. Sci. USA.

[34] Hemnani T, Parihar MS (1998). Reactive oxygen species and oxidative DNA damage. Indian J. Physiol. Pharmacol..

[35] Winterbourn CC (1995). Toxicity of iron and hydrogen peroxide: the Fenton reaction. Toxicol. Lett..

[36] Shan X, Li S, Sun B, Chen Q, Sun J, He Z (2020). Ferroptosis-driven nanotherapeutics for cancer treatment. J. Control. Release.

[37] Zeng C, Tang H, Chen H, Li M, Xiong D (2020). Ferroptosis: a new approach for immunotherapy. Cell Death Discov..

[38] Shan X, Li S, Sun B, Chen Q, Sun J, He Z (2020). Ferroptosis-driven nanotherapeutics for cancer treatment. J .Control. Release.

[39] Conrad M, Kagan VE, Bayir H, Pagnussat GC, Head B, Traber MG (2018). Regulation of lipid peroxidation and ferroptosis in diverse species. Genes Dev..

[40] Bachhawat AK, Ganguli D, Kaur J, Kasturia N, Thakur A, Kaur H (2009). Glutathione production in yeast. Yeast Biotechnol. Divers. Appl..

[41] Manfredini V, Roehrs R, Peralba MC, Henriques JA, Saffi J, Ramos AL (2004). Glutathione peroxidase induction protects *Saccharomyces cerevisiae* sod1deltasod2delta double mutants against oxidative damage. Braz. J. Med. Biol. Res..

[42] Kho CW, Lee PY, Bae KH, Cho S, Lee ZW, Park BC (2006). Glutathione peroxidase 3 of *Saccharomyces cerevisiae* regulates the activity of methionine sulfoxide reductase in a redox state-dependent way. Biochem. Biophys. Res. Commun..

